# Deciphering Immunotoxicity in Animal-Derived Biomaterials: A Genomic and Bioinformatics Approach

**DOI:** 10.3390/ijms252010963

**Published:** 2024-10-11

**Authors:** Huan Lian, Yu Liu, Linnan Ke, Qianqian Han

**Affiliations:** Department of Medical Devices, National Institutes for Food and Drug Control, Beijing 102629, China; lianhuan@nifdc.org.cn (H.L.); liuyu6235@163.com (Y.L.); kelinnan@nifdc.org.cn (L.K.)

**Keywords:** biomaterials, immunotoxicity, genomic analysis, preclinical testing

## Abstract

Immunotoxicity evaluation has been crucial in preclinical testing for implantable animal-derived biomaterials due to their prolonged contact with the human body, which requires stringent safety assessments. By creating experimental models with varying levels of immunotoxicity, this study reveals the decisive role of decellularization treatment in diminishing the immunogenicity of materials, thus ensuring clinical safety. Employing cutting-edge differential gene expression analysis, the research not only accurately quantifies gene expression alterations in immune responses but also, through pathway enrichment analysis, identifies gene networks associated with oncogenesis. This offers novel insights into the mechanisms of immune responses following biomaterial implantation. Additionally, the study highlights the importance of developing highly sensitive immunotoxicity testing methods and validates the efficacy of high-throughput sequencing and bioinformatics tools in assessing biomaterial safety, providing robust scientific support for future preclinical evaluations.

## 1. Introduction

Tissue engineering has introduced innovative solutions for regenerative medicine and organ reconstruction, with animal-derived natural materials being extensively utilized in medical devices [1]. However, a significant concern with the use animal-derived materials is their immunotoxicity to the human body. ISO10993.20 offers guidelines for immune responses and immunotoxicity reactions associated with the implantation of such medical devices, but standardized testing methods are lacking. Given the complexity of the immune system, a multi-test strategy is commonly employed in preclinical testing to gather comprehensive data [2,3].

However, inconsistencies in these assays have resulted in uncertainties regarding material safety. Except for materials that elicit severe immunotoxic phenotypes, most exhibit only mild responses in functional tests [4]. Furthermore, contemporary medical regulations demand a deeper understanding of the mechanisms underlying human interactions with medical devices. Traditional tests have not sufficed in this regard, but genomic analysis has provided new insights. Due to the intricate nature of the immune system and its regulatory network, genomic analysis is more appropriate for elucidating these mechanisms. Techniques such as RT-PCR and in situ nucleic acid hybridization have been effectively applied to evaluate material biocompatibility [5,6]. However, these methods focus on a limited set of well-recognized genes, necessitating researchers to have substantial background knowledge for target gene selection. It is also important to recognize that organisms can maintain equilibrium by managing minor fluctuations in gene expression. These experiments are labor-intensive and challenging to standardize, limiting their widespread application. The rapid advancement of deep sequencing and bioinformatics has enabled the extraction of vast datasets and the revelation of underlying mechanisms at a manageable cost and resource investment.

Molecular markers, characterized by their specific chemical structures and detectable in organisms, serve as crucial indicators for assessing immunotoxicity in preclinical testing [7,8]. In China, regulatory science underscores the imperative to enhance testing methodologies, foster the development and application of biomarker and omics technologies, and establish a novel framework for comprehensively evaluating medical devices [9]. Despite this, relevant research remains scarce. This study employs genomics and diverse bioinformatics approaches to identify key driver genes associated with the immunotoxicity of animal-derived biomaterials. These genes, which may be of significant biological importance, could potentially regulate immune responses following the biomaterial implantation. They may also function as molecular markers of immunotoxicity, assisting in the evaluation of post-implantation immune responses. Gaining insights into their functions could inform the design and refinement of biomaterials to mitigate immune reactions and improve biocompatibility. Moreover, these genes could serve as biomarkers for assessing the safety of biomaterials, thereby substantiating their safety and efficacy.

## 2. Results 

Omics testing is a sensitive and informative method for identifying biomaterials with immunotoxic potential, showing a strong correlation with cytometric bead array (CBA) results. The identified key driver genes may serve as potential molecular markers of immunotoxicity.

### 2.1. Cytometric Bead Array (CBA) Analysis

**Figure 1:**
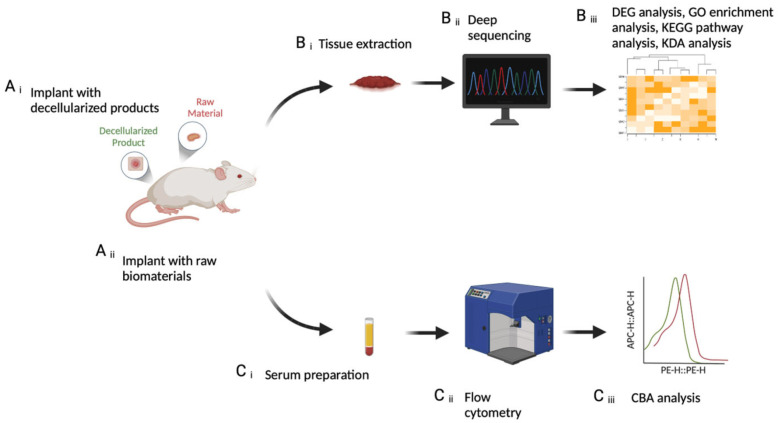
Schematic diagram. (**A**) Subcutaneous implantation of (**i**) decellularized products and (**ii**) raw materials. (**B**) Immunomics pipeline includes (**i**) tissue extraction, (**ii**) deep sequencing, and (**iii**) bioinformatics analysis. (**C**) The CBA pipeline comprises (**i**) serum preparation, (**ii**) flow cytometry, and (**iii**) CBA analysis.

**Figure 2:**
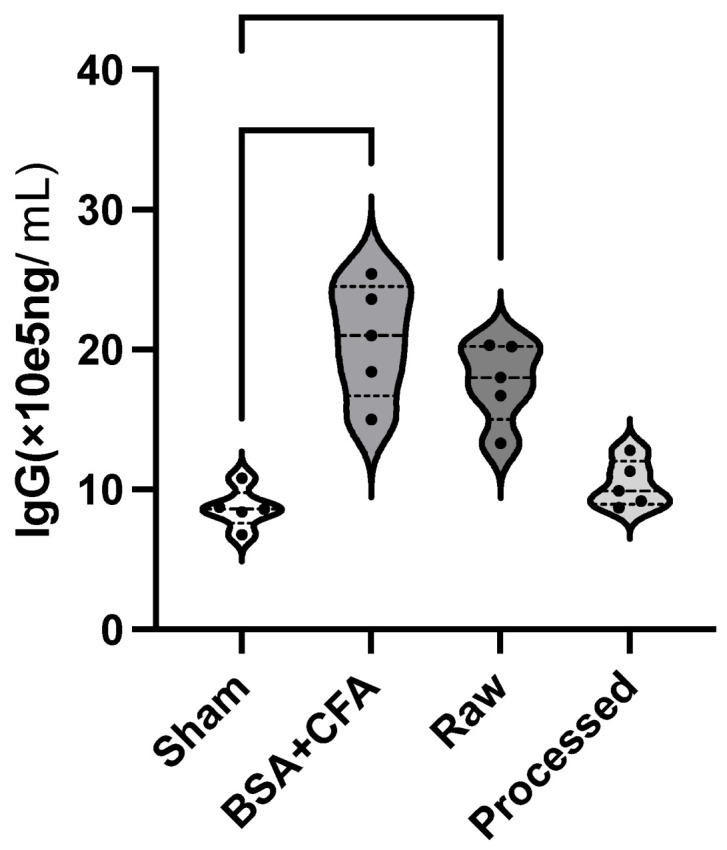
Serum IgG concentration. The *X*-axis represents different experimental groups, and the *Y*-axis represents the IgG concentration.

Twenty mice were randomly assigned into four test groups: positive control, sham operation, test Group 1, and test Group 2. Mice in test Groups 1 and 2 were implanted with raw dermal matrix materials and decellularized dermal matrix materials, respectively (Figure 1A(i,ii)). The positive control group, to ascertain the test system’s sensitivity, received subcutaneous injections of bovine serum albumin (BSA; Solarbio; Beijing, China) mixed with complete Freund’s adjuvant (CFA; Sigma; Shanghai, China) and were immunized on Days 1, 14, and 21 [10]. Group details and treatments are shown in Table 1. Raw materials are derived from pig skin dermal matrices, while decellularized materials are processed from raw materials after decellularization and virus inactivation. The IgG antibody levels in both the positive control and the raw material implantation group exhibited significant immunotoxicity, being more than twice as high as in the sham group (*p* < 0.05) (Figure 2) [11].

### 2.2. Differentially Expressed Gene (DEG) Analysis

**Figure 3:**
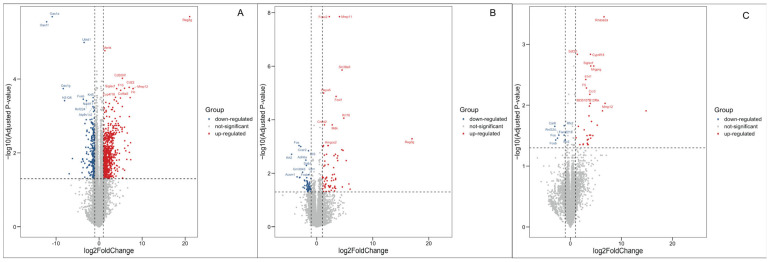
Volcano plots of DEGs. (**A**) The volcano plot displays DEGs between the positive and sham groups. (**B**) The volcano plot shows DEGs between the raw material implantation group and the sham group. (**C**) The volcano plot displays DEGs between the decellularized product implantation group and the sham group. The *X* axis represents the log2-transformed fold change, and the *Y* axis represents the log10-transformed adjusted *p*-value. Red dots indicate up-regulated DEGs, blue dots indicate down-regulated DEGs, and gray dots indicate non-DEGs.

Sequencing results revealed 960 DEGs in the positive control group (Figure 3A), 126 DEGs in the raw material implantation group (Figure 3B), and 40 DEGs in the decellularized product implantation group compared to the sham group (Figure 3C), as detailed in Table 2, Tables S1–S4, and Supplementary Figure S1. As expected, BSA mixed with CFA and raw materials induced a more severe immunotoxic response, with a substantial increase in the number of responsive genes.

### 2.3. DEG Pathway Enrichment Analyses

**Figure 4:**
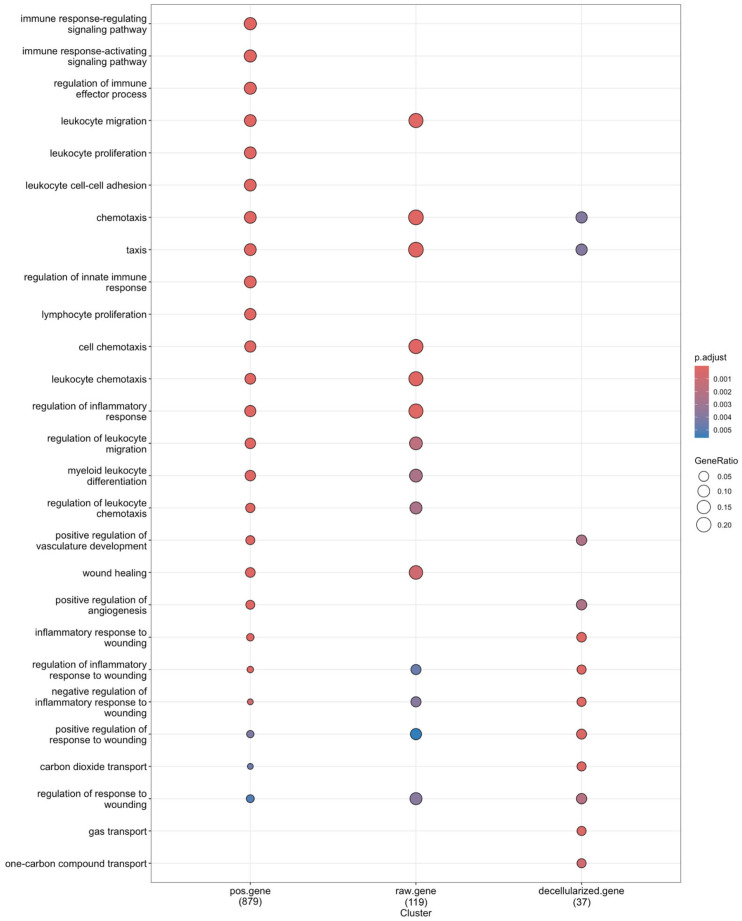
The results of biological pathway enrichment analysis of DEGs. GO enrichment results of the positive group, the raw material implantation group, and the decellularized product implantation group. The “Gene Ratio” on the *X*-axis typically indicates the proportion of genes enriched in specific biological functions or pathways relative to the total gene count. This ratio is utilized to evaluate the statistical significance of the enrichment of the observed gene set within a particular function or pathway. The “Gene Ratio” is calculated as the number of genes associated with a specific function or pathway (numerator) divided by the total number of genes in the enrichment analysis (denominator). An increased ratio suggests a higher likelihood of the function or pathway being biologically significant. The *Y*-axis represented GO_BP annotations. The enriched DEGs are denoted by circles sized according to the number of enriched genes, with color-coding indicating the adjusted *p*-values: red signifies a greater enrichment significance.

**Figure 5:**
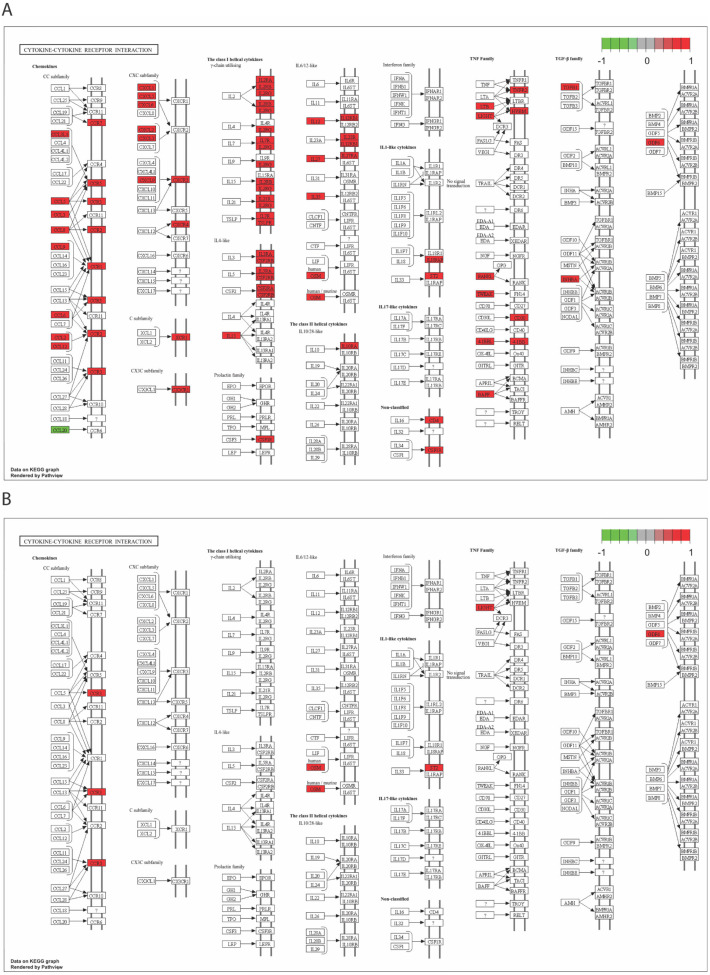
KEGG pathway enrichment analysis of cytokine–cytokine receptor interaction. (**A**) The KEGG pathway enrichment plot for the positive group. (**B**) The KEGG pathway enrichment plot for the raw material implantation group. Red indicates up-regulated genes, and green indicates down-regulated genes.

The DEGs obtained from the positive control group, the raw material implantation group, and the decellularized product implantation group were categorized based on the biological process (BP) sub-class of the gene ontology (GO) database and Kyoto Encyclopedia of Genes and Genomes (KEGG) pathway databases [12,13]. The majority of DEGs in the positive control group and raw material implantation group were found to be enriched in immune signaling pathways and chemotaxis processes. Notably, chemotaxis processes were particularly prominent in the raw material implantation group. However, the DEGs of the decellularized product implantation group were predominantly associated with wound-healing processes rather than active immune processes (Figure 4, Supplementary Figure S2 and Tables S5–S7). Furthermore, for shared biological processes, such as the cytokine–cytokine receptor interaction process, the positive control group and the raw material implantation group exhibited varying degrees of activation and distinct pathways (Figure 5). The low immunotoxicity of decellularized products led to a limited number of DEGs, which prevented the enrichment of the cytokine–cytokine receptor interaction pathway. To examine the collective expression patterns of the full gene set across defined biological conditions, we integrated Gene Set Enrichment Analysis (GSEA) into our study. These findings align with those of the enrichment analysis (Supplementary Figure S3) [14].

### 2.4. Protein–Protein Interaction (PPI) Network Construction and Key Driver Gene Analysis (KDA)

**Figure 6:**
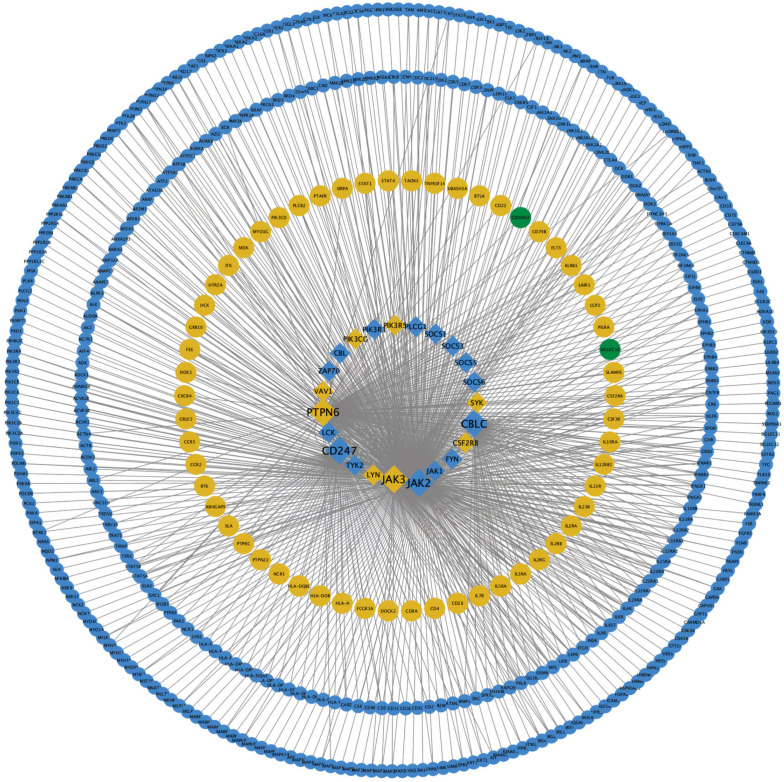
PPI network and key driver gene identification. Rhomboids represent identified key driver genes; circles represent genes directly interacting with key driver genes. Larger sizes indicate higher scores. Colors denote DEG sources: blue for the positive group; yellow for the raw material implantation group; and green for the decellularized product implantation group.

Upon analysis with KDA, the top 10 driver genes were identified (Table 3 and Figure 6). These include *PTPN6*, *JAK2*, *CBLC*, *CD247*, *JAK3*, *JAK1*, *SOCS1*, *LCK*, *SOCS6*, and *CBL* in a descending order. Notably, *PTPN6,* along with three other proto-oncogenes, is a gene of considerable interest in immunology and oncology [15,16,17,18]. Additionally, genes associated with cytokine signaling were significantly enriched, particularly those within the JAK/STAT pathway and the SOCS family [19,20]. CD247, predominantly expressed in natural killer (NK) cells and T cells, was also identified. It plays a pivotal role in the assembly and activation of the T cell receptor complex [21].

## 3. Discussion

Immunotoxicity testing is central to the evaluation of biocompatibility, yet its methods have seen minimal updates since the early 20th century. Current test protocols necessitate a comprehensive analysis of data by researchers. Furthermore, multiple dosages are assessed to discern dose-dependent effects. However, medical devices differ from chemical drugs, often eliciting milder toxic reactions in some tests, which could lead to false negatives [22,23]. Consequently, we have explored a pipeline that integrates deep sequencing and bioinformatics to establish a more sensitive and systematic approach for analyzing the immunotoxicity of medical devices.

Molecular markers of immunotoxicity, indicative of immune cell activity and immune response status, are crucial for diagnosing, treating, and prognostically assessing immune-related diseases. Monitoring these markers enhances our ability to evaluate animal-derived biomaterials in preclinical testing and to predict patient treatment outcomes. In addition, genomic analysis can elucidate the mechanism of interaction between medical devices and biological systems, thereby complementing the existing preclinical standards and product evaluation methods.

Preclinical testing aims to ensure product safety prior to human application and not to incur additional costs or impose unnecessary hurdles to industry development. Therefore, it may be more practical to design microarray chips for key driver genes and associated initial genes identified in this study. The established results and testing methods offer a novel perspective for immunotoxicity testing in medical devices, thereby enhancing the associative testing standardization system. However, it is imperative to recognize that the introduction of new methods will also present new challenges to the research, production, and regulatory bodies in the field. Therefore, further validation of the selected targets is essential.

## 4. Materials and Methods

### 4.1. Materials

Raw materials are dermal matrix materials derived from porcine skin. Decellularized dermal matrix materials are prepared by the decellularization and virus inactivation of raw materials. The primary constituent is collagen, which forms a porous, three-dimensional network. The materials are sterilized by electron beam irradiation for single-use applications.

### 4.2. Experimental Animals

Healthy BALB/c mice (*N* = 20, aged 6–8 weeks, weighing 20 ± 2 g) were procured from the Animal Breeding Center of China Research Institute of Food and Drug Control (Beijing, China). All mice were housed in specific pathogen-free conditions at 18–22 °C, with a relative humidity of 40–70%, and a 12 h light/dark cycle. All animal protocols described herein were approved by the Ethics Committee of National Institutes for Food and Drug Control (Ethics Code #2019 (A)043).

### 4.3. Subcutaneous Implantation

Prior to surgery, mice were anesthetized with an injection of zoletil at a dosage of 50 mg/kg (Virbac, Kavenan, France). Once anesthetized, the skin along both sides of the spine was disinfected and shaved, and an incision was made to facilitate the implantation of a subcutaneous capsule. Mice in the sham operation group underwent a surgical procedure without the implantation of any materials.

### 4.4. CBA Analysis

Whole blood samples were collected in EP tubes, set still for 2 hr, and then centrifuged at 3500 rpm for 10 min. A 100 μL aliquot from each serum sample was collected and diluted 10,000-fold using phosphate-buffered saline (PBS).For the assay, 50 μL aliquots of diluted blood serum from mice implanted with either raw or decellularized materials, as well as from control groups, were added to individual tubes. Each tube was then supplemented with 50 μL of a solution containing IgG microbeads (BD Biosciences, San Jose, CA, USA). The tubes were incubated at room temperature for 15 min with gentle shaking. Following incubation, all tubes were washed with 1 mL PBS and centrifuged at 1000 rpm for 30 min. The microbeads were collected and resuspended with 100 μL PBS. Then, 50 μL PE of PE/FITC solution was added to each tube. After incubation in the dark at room temperature for 15 min, all tubes were washed and resuspended with 500 μL PBS. The IgG antibody levels were measured with flow cytometry on an FACSVerse flow cytometry system (BD Biosciences, San Jose, CA, USA) with the associated system software (Figure 1C(i–iii)) [24].

### 4.5. Tissue Extraction

The skin at the implantation site on the mice’s dorsal surfaces was excised and promptly rinsed with normal saline to eliminate blood and debris (Figure 1B(i)). Subsequently, non-essential connective tissues were excised, and excess fluid was drained. The tissues were then sectioned into approximately 50 mg pieces and immediately cryopreserved in liquid nitrogen. All samples were stored at −80 °C until they were dispatched to the sequencing facility for cold chain transportation.

### 4.6. DEG Analysis

The quality of raw data was assessed using fastQC 0.12.0 software [25]. After filtering out low-expression genes, DEGs were analyzed with the RStudio 4.4.1 software package DESeq2 [26]. The DEGs were identified using a criterion of an absolute log2 ford change (log2 FC) of greater than 1 and an adjusted *p*-value of ≤ 0.05. Data visualization was conducted using the ggplot2 library in the RStudio 4.4.1 software [27]. Genes with Log2 FC > 1 were classified as up-regulated, while those with log2 FC < −1 were considered down-regulated.

### 4.7. DEG Pathway Enrichment Analyses

The clusterProfiler package in RStudio 4.4.1 was applied to study the biological functions of DEGs [28]. The enrichment analysis was conducted based on GO-BP and KEGG databases, with *p*-value and q-value thresholds set at 0.05. The pathview package was employed to visualize the KEGG pathway enrichment outcomes [29].

### 4.8. PPI Network Construction and KDA

After merging the DEGs of the three groups, we employed Mergeomics 2.0 software to analyze the PPI network of these DEGs [30]. Mergeomics is a flexible and user-friendly online tool for analyzing multi-omics data to uncover biological pathways, networks, and key drivers essential for disease pathogenesis. It utilizes the open-source R package and efficiently processes summary statistics from various omics studies, including GWAS, EWAS, TWAS, and PWAS. The systematic identification of key drivers involves initial data input, where users submit relevant omics summary statistics, gene marker mappings, gene sets, and regulatory networks. Users can then tailor the analysis by setting parameters such as permutation types and gene number thresholds. The analysis proceeds with Mergeomics employing Molecular Set Enrichment Analysis (MSEA) to detect significant gene sets, which are integrated with gene networks for KDA to identify key genes. The outcome presents these genes within specific networks, highlighting their biological significance and providing insights into disease mechanisms. The resulting PPI network was visualized using the Cytoscape v3.10.2 software [31]. Thereafter, the CytoHubba module was applied to compute the maximal clique centrality (MCC) between genes, and the top 10 key driver genes were identified according to the MCC score [32].

## 5. Conclusions

Animal-derived biomaterials, extensively utilized in medical devices such as valves and biological patches, necessitate the preclinical assessment of immunotoxicity due to their prolonged contact with the human body. This study categorized three groups exhibiting varying levels of immunotoxicity. BSA+CFA serves as a standard positive control in immunotoxicity assays, demonstrating potent immunotoxic effects. Conversely, pig skin tissue in its raw form exhibits significant immunotoxicity in clinical settings. The decellularization process effectively removes most antigenic components, rendering the final products suitable for clinical use. CBA results corroborated this finding, yet they fail to discern the intensity of immunotoxicity between the positive control group and the raw material implantation group. Thus, traditional testing methods could not yield critical insights into the degree of immunotoxicity.

However, the analyses of DEGs reveals that the positive agent group has the highest number of DEGs, followed by the raw material implantation group, with the decellularized product implantation group showing the fewest. This pattern allows for a preliminary assessment of immunotoxicity, as the quantity and distribution of DEGs can indicate an organism’s response to an immunotoxic stimulus. Concurrently, enrichment analysis, which mirrors this trend, provides additional insights into the signaling pathways involved. For instance, the positive group shows enrichment in immune response signaling and chemotaxis processes, which correlates with the significant increase in IgG levels observed in CBA results. In contrast, the raw material implantation group notably enriches the chemotaxis process, and the wound healing process is enriched due to surgical intervention. The enriched wound healing and repair processes in the decellularized product implantation group are consistent with the presence of DEGs, aligning with our expectations.

Beyond these distinct enriched processes, we observed varying degrees of activation in shared processes between the positive control and raw material implantation groups. For example, the cytokine–cytokine receptor interaction process is more extensively activated in the positive control group. To thoroughly investigate immunotoxicity-related biomarkers, we integrated the DEGs from all three groups and identified key driver genes by mapping PPIs and calculating MCC scores. In this study, JAK1, JAK2, and JAK3 were identified as driver genes. The JAK-STAT signaling pathway regulates the expression of genes involved in immune responses, cell proliferation, and survival. Consequently, the activation of these JAK genes may alter downstream gene expression, activate immune cells, and establish a positive feedback loop through the JAK-STAT pathway to sustain signal transduction [19]. Additionally, SOCS1 and SOCS6, identified as key genes, may modulate the JAK-STAT pathway, influencing the activation, proliferation, and cytotoxicity of natural killer (NK) cells, thus affecting immune responses. Importantly, aberrant SOCS protein expression can lead to continuous JAK-STAT pathway activation, promoting tumorigenesis and tumor progression [20]. CBL and CBLC, E3 ubiquitin ligases and members of the CBL family, are crucial for signal transduction and receptor tyrosine kinase (RTK) stability. They regulate protein stability and activity in the JAK-STAT pathway and modulate RTK activity and stability, influencing other immune response-related signaling pathways. In tumors, CBL proteins potentially regulate cell proliferation and survival by modulating survival signaling pathways, thereby reinforcing the hypothesis that the implantation of immunotoxic materials elevates the risk of carcinogenesis [17]. LCK, a key tyrosine kinase in the T cell receptor (TCR) pathway, initiates TCR/CD3 complex phosphorylation, triggering downstream signaling. It regulates T cell proliferation, differentiation, and effector functions, directly impacting immune responses mediated by T cells and is regulated by the CBL family [18]. CD247, a TCR-CD3 complex component, is vital for T cell activation and may participate in antigen recognition and response upon exposure to immunogenic materials. The TCR-CD3 complex comprises seven chains, including two ζ chains with three ITAMs that phosphorylate upon T cell activation. Protein kinases like Lck, Fyn, ZAP-70, and other ζ chain-interacting proteins regulate CD247 activity [21]. PTPN6, identified as a predominant driver gene by KDA selection, is crucial for maintaining cellular homeostasis and modulating inflammatory responses. It has been shown to inhibit apoptosis and necroptosis, thereby playing a significant role in cellular equilibrium. It modulates signaling pathways, including inhibiting MyD88, enhancing receptor-interacting protein kinases, and suppressing RIPK3/MLKL necrosis, caspase-8 apoptosis, and p38/MAPK, thus controlling inflammation [15,16].

Therefore, the implantation of immunotoxic materials significantly affects the entire organism, underscoring the importance of developing more sensitive preclinical testing methods. Currently, animal-derived natural materials are extensively utilized in tissue reconstruction medical devices and have garnered significant industry interest [33]. However, the genome-level immunotoxicity testing remains largely unexplored. This study confirms the utility of high-throughput sequencing and bioinformatics tools in immunotoxicity assessments, potentially elucidating the underlying mechanisms of immune responses following the implantation of biomedical materials. These key gene expressions or activities correlate with immune response characteristics and can serve as biomarkers for immunotoxicity detection in preclinical trials of animal-derived products and for early diagnosis, prognosis assessment, and therapeutic monitoring of diseases.

## Figures and Tables

**Table 1:** Experimental grouping and treatments.

Grouping	Treatment	Dosage
Positive control	2 mg/mL BSA+CFA	0.1 mL
Experimental control	Sham operation	/
Test Group1	Raw material	1.0 cm × 0.5 cm
Test Group 2	Decellularized products	1.0 cm × 0.5 cm

**Table 2:** Quantity of differentially expressed genes.

Grouping	Quantity	
	Up	Down
Positive vs. sham	707	253
Raw vs. sham	68	58
Processed vs. sham	33	7

**Table 3:** Top 10 key driver genes.

Rank	Key Driver Node	Gene Product
1	PTPN6	Protein tyrosine phosphatase non-receptor type 6
2	JAK2	Janus kinase 2
3	CBLC	Cbl proto-oncogene C
4	CD247	CD247 molecule
5	JAK3	Janus kinase 3
6	JAK1	Janus kinase 1
7	SOCS1	Suppressor of cytokine signaling 1
8	LCK	LCK proto-oncogene
9	SOCS6	Suppressor of cytokine signaling 6
10	CBL	Cbl proto-oncogene

## Data Availability

Data are available from the corresponding author upon request.

## Supplementary Materials

The following supporting information can be downloaded at: https://www.mdpi.com/article/10.3390/ijms252010963/s1.

## References

[1] Cao D., Ding J. (2022). Recent advances in regenerative biomaterials. Regen. Biomater..

[2] (2006). Biological Evaluation of Medical Devices-Priciples and Methods for Immunotoxicology Testing of Medical Devices.

[3] (2003). Standard Practice for Evaluation of Immune Repsonses in Biocompatibility-Testing Using ELISA Tests, Lymphocyte Proliferation, and Cell Migration.

[4] Lu Q.J. (2007). Current status and challenge of biopharmaceutical immunogenicity. Chin. J. New Drugs.

[5] Crawford L., Wyatt M., Bryers J., Ratner B. (2021). Biocompatibility Evolves: Phenomenology to Toxicology to Regeneration. Adv. Healthc. Mater..

[6] Wiencke J.K., Bracci P.M., Hsuang G., Zheng S., Hansen H., Wrensch M.R., Rice T., Eliot M., Kelsey K.T. (2014). A comparison of DNA methylation specific droplet digital PCR (ddPCR) and real time qPCR with flow cytometry in characterizing human T cells in peripheral blood. Epigenetics.

[7] Jung S.M., Park K.S., Kim K.J. (2022). Integrative analysis of lung molecular signatures reveals key drivers of systemic sclerosis-associated interstitial lung disease. Ann. Rheum. Dis..

[8] Wu B., Xi S. (2021). Bioinformatics analysis of differentially expressed genes and pathways in the development of cervical cancer. BMC Cancer.

[9] Shi J., Lai Y.F., Liang Z., Wang J., Lin H., Li M., Ieong L.C., Hu H., Ung C.O.L. (2020). PNS33 Developing and Adopting Regulatory Science: Experiences of Drug Regulatory Authorities in China, United States, European Union, and JAPAN. Value Health Reg. Issues.

[10] Du X.D., Fang Y., Xi T.F., Wang C.R. (2010). Using BSA as a positive control for cellular immunotesting of animal-derived medical devices. J. Drug Anal..

[11] Cook E., Stahl J., Lowe L., Chen R., Morgan E., Wilson J., Varro R., Chan A., Graziano F.M., Barney N.P. (2001). Simultaneous measurement of six cytokines in a single sample of human tears using microparticle-based flow cytometry: Allergics vs. non-allergics. J. Immunol. Methods.

[12] Ding J., Zhang Y. (2017). Analysis of key GO terms and KEGG pathways associated with carcinogenic chemicals. Comb. Chem. High. Throughput Screen..

[13] Kanehisa M., Furumichi M., Tanabe M., Sato Y., Morishima K. (2017). KEGG: New perspectives on genomes, pathways, diseases and drugs. Nucleic Acids Res..

[14] Subramanian A., Tamayo P., Mootha V.K., Mukherjee S., Ebert B.L., Gillette M.A., Paulovich A., Pomeroy S.L., Golub T.R., Lander E.S. (2005). Gene set enrichment analysis: A knowledge-based approach for interpreting genome-wide expression profiles. Proc. Natl. Acad. Sci. USA.

[15] Wu N., Sun Y., Xue D., He X. (2024). FTO promotes the progression of bladder cancer via demethylating m6A modifications in PTPN6 mRNA. Heliyon.

[16] Kiratikanon S., Chattipakorn S.C., Chattipakorn N., Kumfu S. (2022). The regulatory effects of PTPN6 on inflammatory process: Reports from mice to men. Arch. Biochem. Biophys..

[17] Ren J., Lv L., Tao X., Zhai X., Chen X., Yu H., Zhao X., Kong X., Yu Z., Dong D. (2024). The role of CBL family ubiquitin ligases in cancer progression and therapeutic strategies. Front. Pharmacol..

[18] Wu J., Li G., Li L., Li D., Dong Z., Jiang P. (2021). Asparagine enhances LCK signalling to potentiate CD8^+^ T-cell activation and anti-tumour responses. Nat. Cell Biol..

[19] Banerjee S., Biehl A., Gadina M., Hasni S., Schwartz D.M. (2017). JAK-STAT Signaling as a Target for Inflammatory and Autoimmune Diseases: Current and Future Prospects. Drugs.

[20] Keating N., Nicholson S.E. (2019). SOCS-mediated immunomodulation of natural killer cells. Cytokine.

[21] Dexiu C., Xianying L., Yingchun H., Jiafu L. (2022). Advances in CD247. Scand. J. Immunol..

[22] Galili U., LaTemple D.C., Radic M.Z. (1998). A sensitive assay for measuring alpha-Gal epitope expression on cells by a monoclonal anti-Gal antibody. Transplantation.

[23] Lu Y., Shao A., Shan Y., Zhao H., Leiguo M., Zhang Y., Tang Y., Zhang W., Jin Y., Xu L. (2018). A standardized quantitative method for detecting remnant alpha-Gal antigen in animal tissues or animal tissue-derived biomaterials and its application. Sci. Rep..

[24] Sprokholt J.K., Hertoghs N., Geijtenbeek T.B. (2016). Flow Cytometry-Based Bead-Binding Assay for Measuring Receptor Ligand Specificity. Methods Mol. Biol..

[25] Wingett S.W., Andrews S. (2018). FastQ Screen: A tool for multi-genome mapping and quality control. F1000Research.

[26] Love M.I., Huber W., Anders S. (2014). Moderated estimation of fold change and dispersion for RNA-seq data with DESeq2. Genome Biol..

[27] Ito K., Murphy D. (2013). Application of ggplot2 to Pharmacometric Graphics. CPT Pharmacomet. Syst. Pharmacol..

[28] Wu T., Hu E., Xu S., Chen M., Guo P., Dai Z., Feng T., Zhou L., Tang W., Zhan L. (2021). clusterProfiler 4.0: A universal enrichment tool for interpreting omics data. Innovation.

[29] Luo W., Brouwer C. (2013). Pathview: An R/Bioconductor package for pathway-based data integration and visualization. Bioinformatics.

[30] Ding J., Blencowe M., Nghiem T., Ha S.M., Chen Y.W., Li G., Yang X. (2021). Mergeomics 2.0: A web server for multi-omics data integration to elucidate disease networks and predict therapeutics. Nucleic Acids Res..

[31] Shannon P., Markiel A., Ozier O., Baliga N.S., Wang J.T., Ramage D., Amin N., Schwikowski B., Ideker T. (2003). Cytoscape: A software environment for integrated models of biomolecular interaction networks. Genome Res..

[32] Ma Z., Zhong P., Yue P., Sun Z. (2023). Identification of immune-related molecular markers in intracranial aneurysm (IA) based on machine learning and cytoscape-cytohubba plug-in. BMC Genom. Data.

[33] Brown M., Li J., Moraes C., Tabrizian M., Li-Jessen N.Y.K. (2022). Decellularized extracellular matrix: New promising and challenging biomaterials for regenerative medicine. Biomaterials.

