# Supplementary material for: Deciphering Immunotoxicity in Animal-Derived Biomaterials: A Genomic and Bioinformatics Approach
Source: Int J Mol Sci. 2024 Oct 11;25(20):10963. doi: 10.3390/ijms252010963 (PMC11507131; doi:10.3390/ijms252010963)
Supplement: Supplementary file 1 [file ijms-25-10963-s001.zip › Supplementary Figures.pdf]

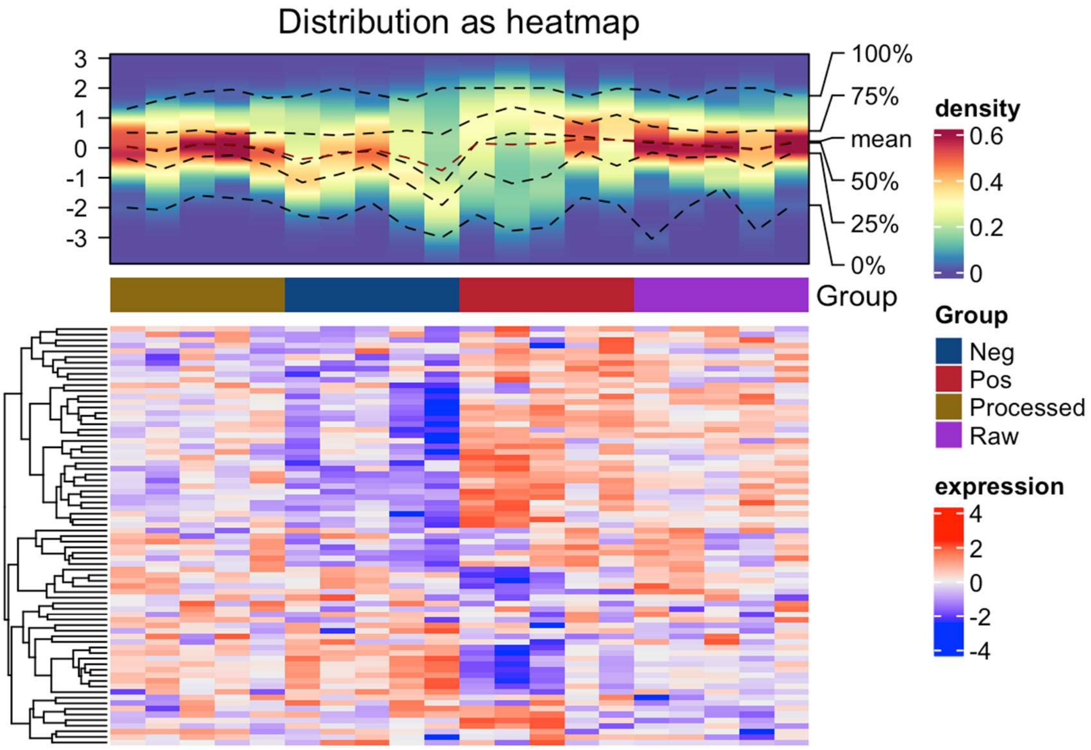

Supplementary Figure S1. Heatmap for Top100 key driver genes in all 4 groups.

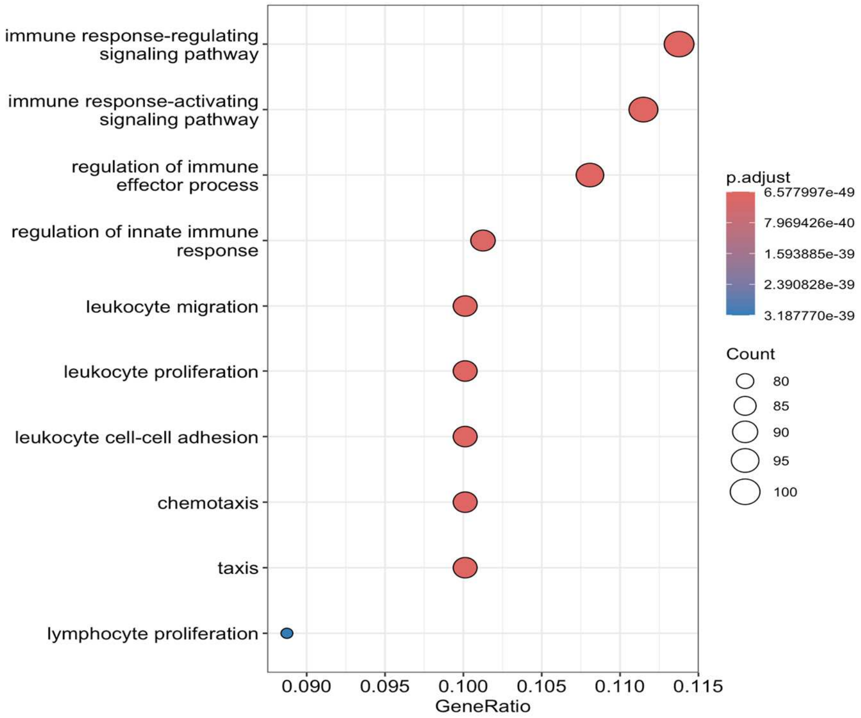

(a)

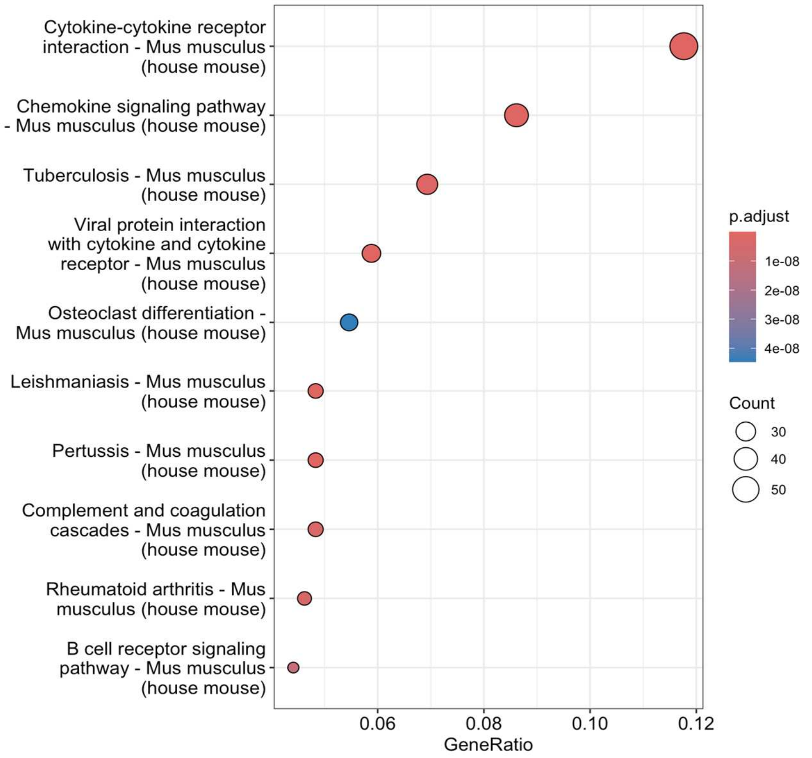
Cytokine-cytokine receptor interaction - Mus musculus (house mouse)
Chemokine signaling pathway - Mus musculus (house mouse)
Tuberculosis - Mus musculus (house mouse)
Viral protein interaction with cytokine and cytokine receptor - Mus musculus (house mouse)
Osteoclast differentiation - Mus musculus (house mouse)
Leishmaniasis - Mus musculus (house mouse)
Pertussis - Mus musculus (house mouse)
Complement and coagulation cascades - Mus musculus (house mouse)
Rheumatoid arthritis - Mus musculus (house mouse)
B cell receptor signaling pathway - Mus musculus (house mouse)
0.06
0.08
0.10
0.12
GeneRatio
p.adjust
1e-08
2e-08
3e-08
4e-08
Count
30
40
50

(b)

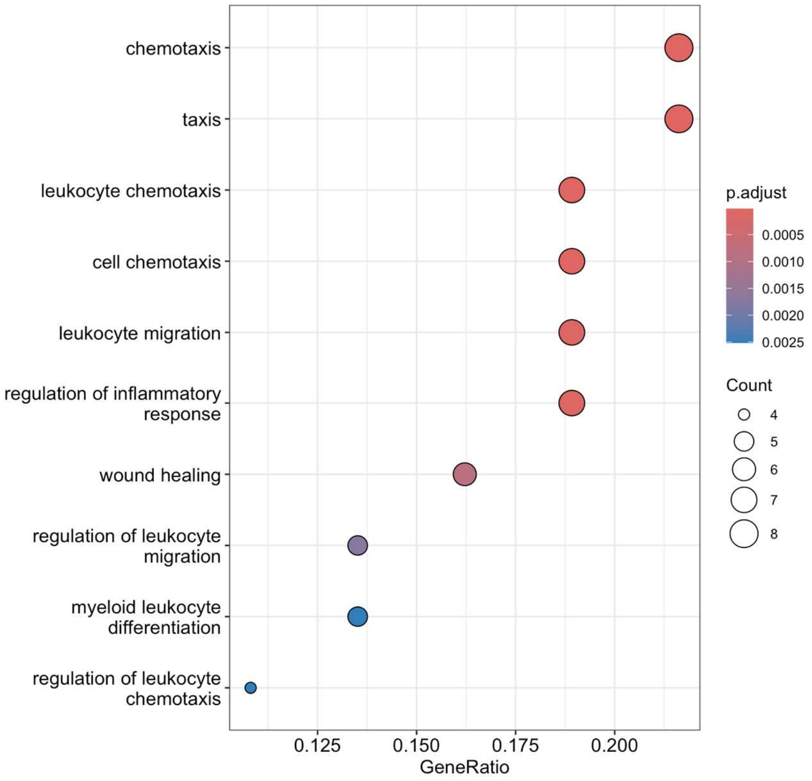
chemotaxis
taxis
leukocyte chemotaxis
cell chemotaxis
leukocyte migration
regulation of inflammatory response
wound healing
regulation of leukocyte migration
myeloid leukocyte differentiation
regulation of leukocyte chemotaxis
0.125
0.150
0.175
0.200
GeneRatio
p.adjust
0.0005
0.0010
0.0015
0.0020
0.0025
Count
4
5
6
7
8

(c)

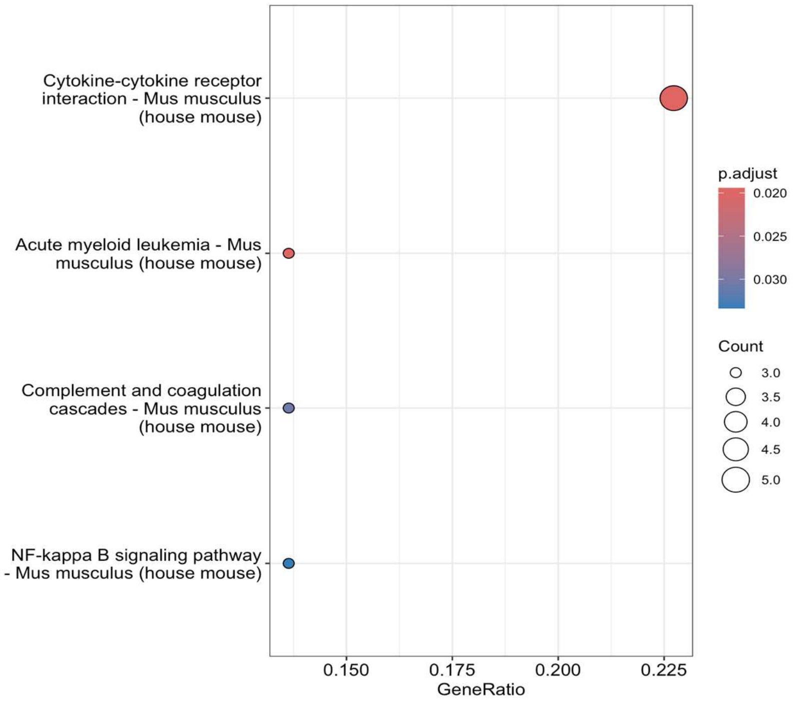
Cytokine-cytokine receptor interaction - Mus musculus (house mouse)
Acute myeloid leukemia - Mus musculus (house mouse)
Complement and coagulation cascades - Mus musculus (house mouse)
NF-kappa B signaling pathway - Mus musculus (house mouse)
0.150
0.175
0.200
0.225
GeneRatio
p.adjust
0.020
0.025
0.030
Count
3.0
3.5
4.0
4.5
5.0

(d)

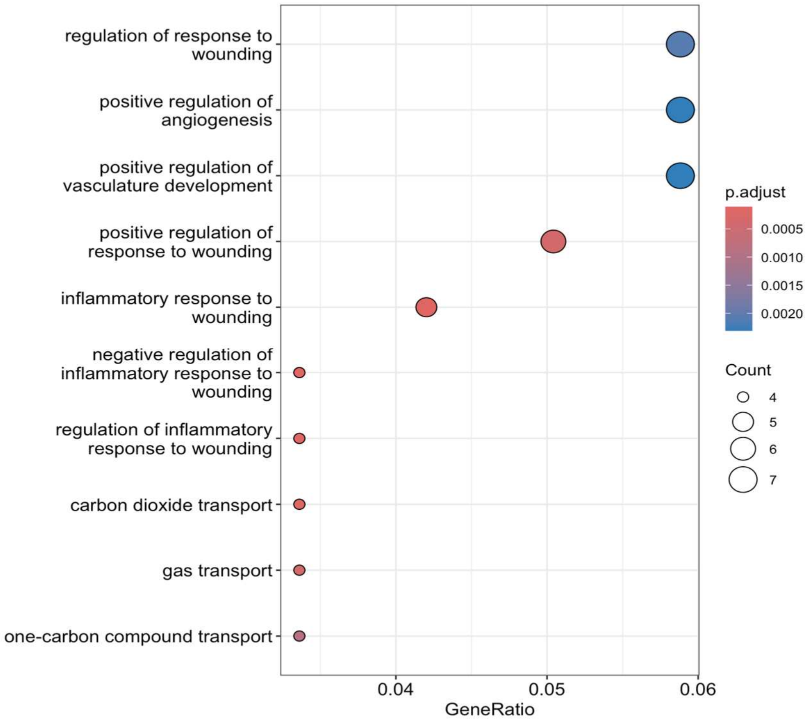
regulation of response to wounding
positive regulation of angiogenesis
positive regulation of vasculature development
positive regulation of response to wounding
inflammatory response to wounding
negative regulation of inflammatory response to wounding
regulation of inflammatory response to wounding
carbon dioxide transport
gas transport
one-carbon compound transport
0.04
0.05
0.06
GeneRatio
p.adjust
0.0005
0.0010
0.0015
0.0020
Count
4
5
6
7

(e)

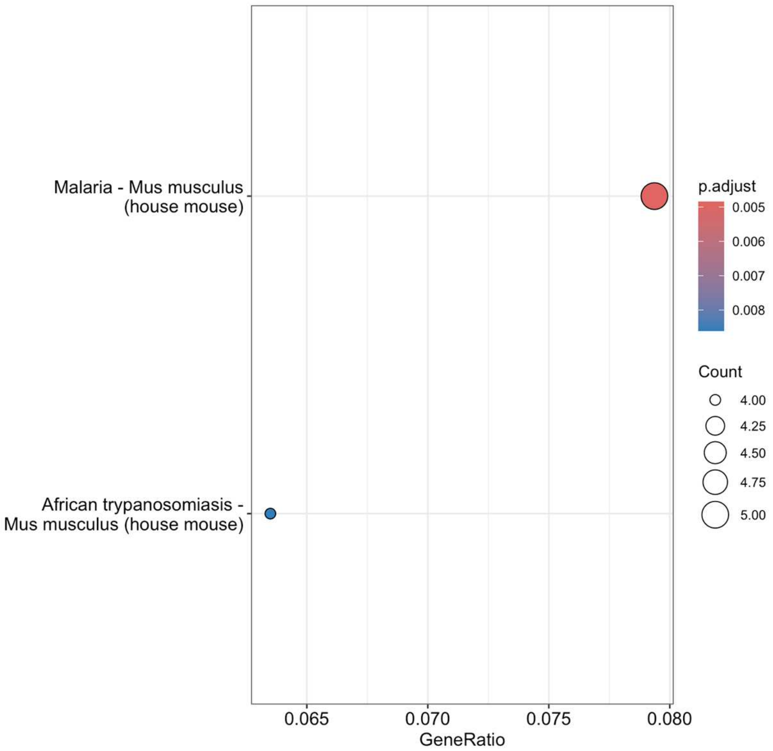

(f)

Supplementary Figure S2. (a) GO-BP enrichment_Pos_vs_Neg; (b) KEGG enrichment_Pos_vs_Neg; (c) GO-BP enrichment_Raw_vs_Neg; (d) KEGG enrichment_Raw_vs_Neg; (e) GO-BP enrichment_Processed_vs_Neg; (f) KEGG enrichment_Processed_vs_Neg.

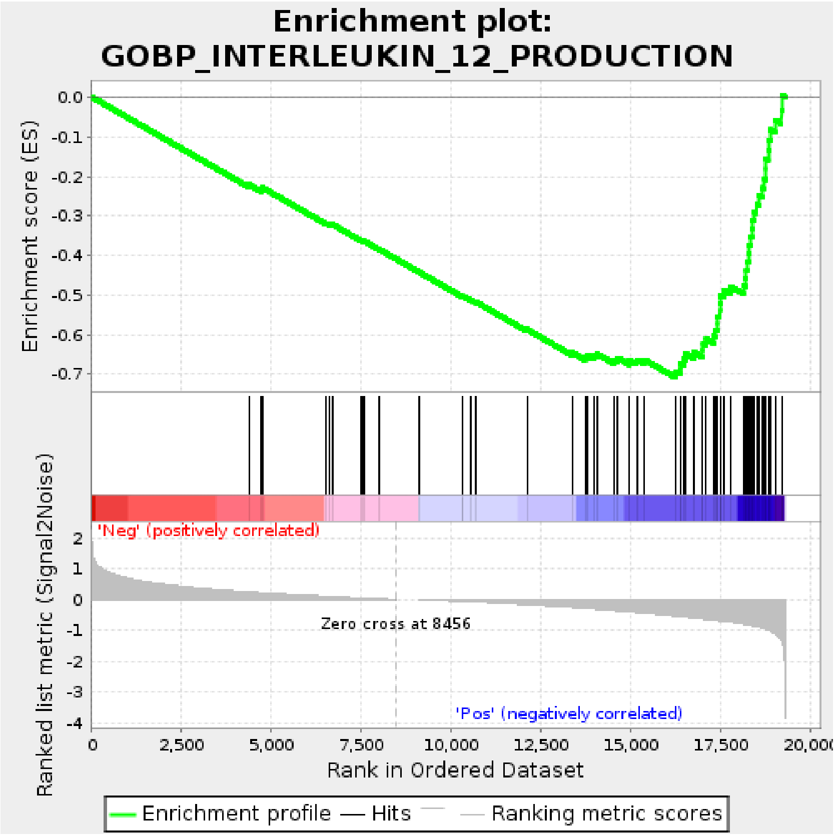
Enrichment plot:
GOBP_INTERLEUKIN_12_PRODUCTION
Enrichment score (ES)
'Neg' (positively correlated)
Zero cross at 8456
'Pos' (negatively correlated)
Ranked list metric (Signal2Noise)
Rank in Ordered Dataset
Enrichment profile — Hits — Ranking metric scores

(a)

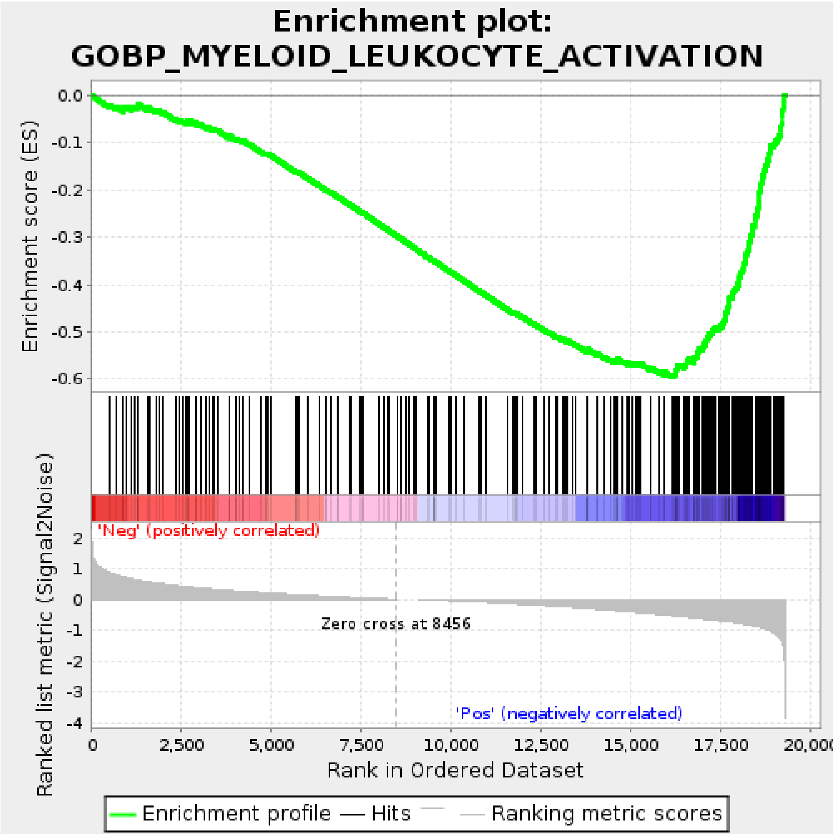
Enrichment plot:
GOBP_MYELOID_LEUKOCYTE_ACTIVATION
Enrichment score (ES)
'Neg' (positively correlated)
Zero cross at 8456
'Pos' (negatively correlated)
Ranked list metric (Signal2Noise)
Rank in Ordered Dataset
Enrichment profile — Hits — Ranking metric scores

(b)

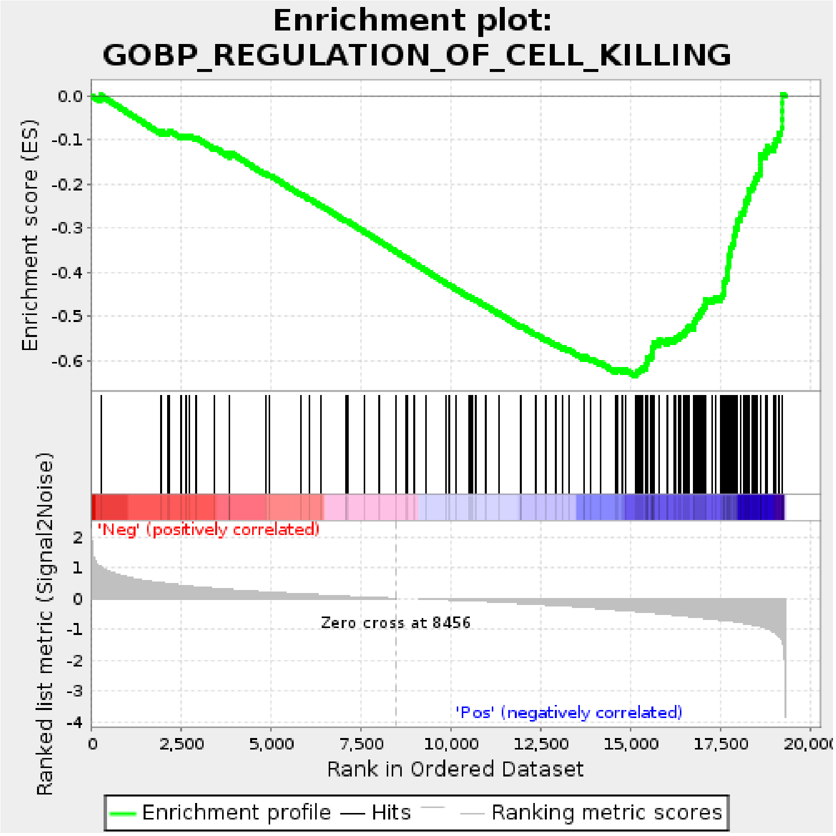
Enrichment plot:
GOBP_REGULATION_OF_CELL_KILLING
Enrichment score (ES)
Ranked list metric (Signal2Noise)
'Neg' (positively correlated)
Zero cross at 8456
'Pos' (negatively correlated)
Rank in Ordered Dataset
Enrichment profile — Hits — Ranking metric scores

(c)

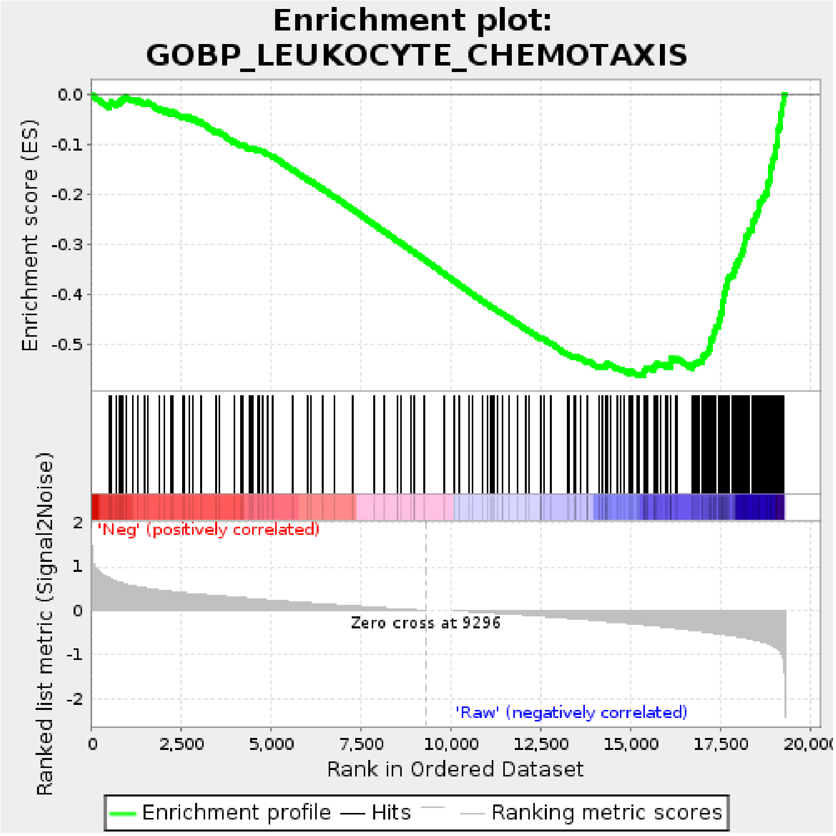
Enrichment plot:
GOBP_LEUKOCYTE_CHEMOTAXIS
Enrichment score (ES)
Ranked list metric (Signal2Noise)
'Neg' (positively correlated)
Zero cross at 9296
'Raw' (negatively correlated)
Rank in Ordered Dataset
Enrichment profile — Hits — Ranking metric scores

(d)

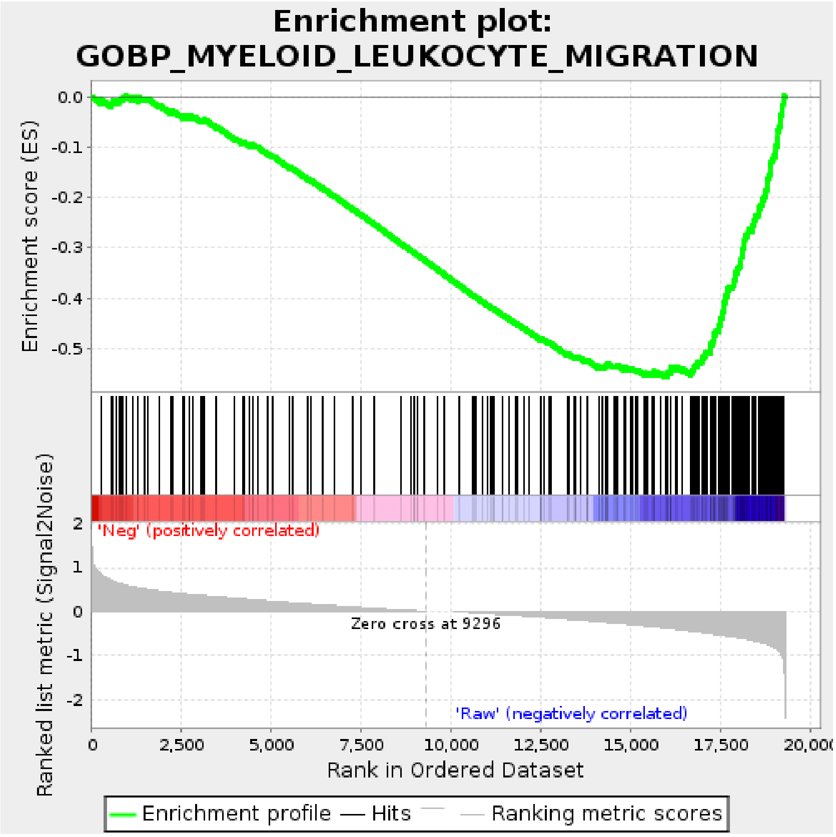

Enrichment plot:
GOBP_MYELOID_LEUKOCYTE_MIGRATION
Enrichment score (ES)
0.0
-0.1
-0.2
-0.3
-0.4
-0.5
Ranked list metric (Signal2Noise)
2
1
0
-1
-2
'Neg' (positively correlated)
Zero cross at 9296
'Raw' (negatively correlated)
0
2,500
5,000
7,500
10,000
12,500
15,000
17,500
Rank in Ordered Dataset
Enrichment profile
Hits
Ranking metric scores

(e)

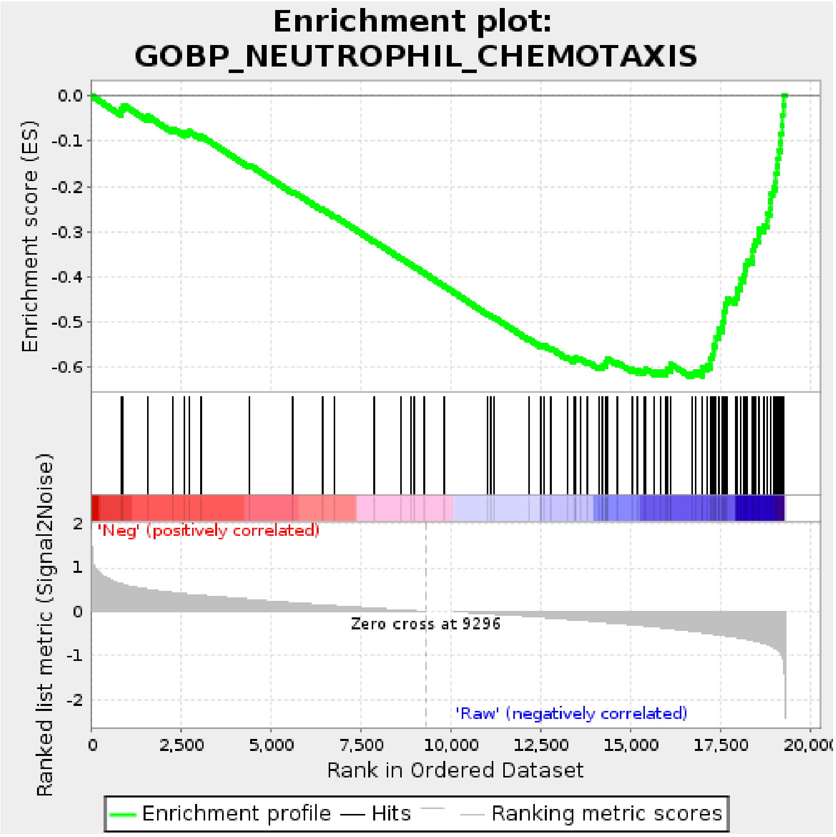

Enrichment plot:
GOBP_NEUTROPHIL_CHEMOTAXIS
Enrichment score (ES)
0.0
-0.1
-0.2
-0.3
-0.4
-0.5
-0.6
Ranked list metric (Signal2Noise)
2
1
0
-1
-2
'Neg' (positively correlated)
Zero cross at 9296
'Raw' (negatively correlated)
0
2,500
5,000
7,500
10,000
12,500
15,000
17,500
Rank in Ordered Dataset
Enrichment profile
Hits
Ranking metric scores

(f)

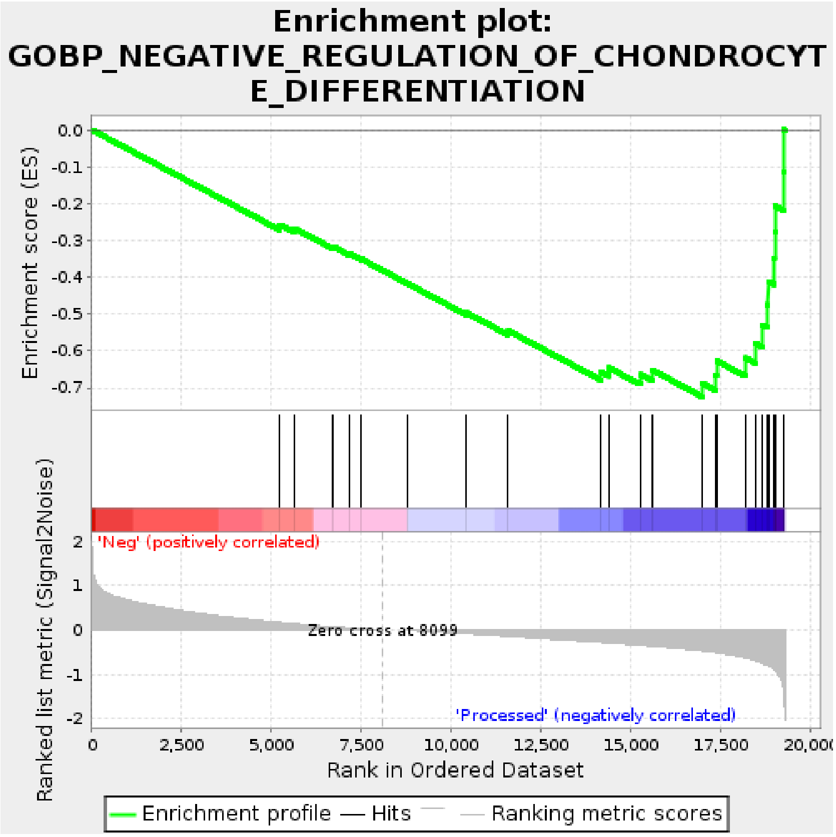
Enrichment plot:
GOBP_NEGATIVE_REGULATION_OF_CHONDROCYTE_DIFFERENTIATION
Enrichment score (ES)
Ranked list metric (Signal2Noise)
'Neg' (positively correlated)
Zero cross at 8099
'Processed' (negatively correlated)
Rank in Ordered Dataset
Enrichment profile — Hits — Ranking metric scores

(g)

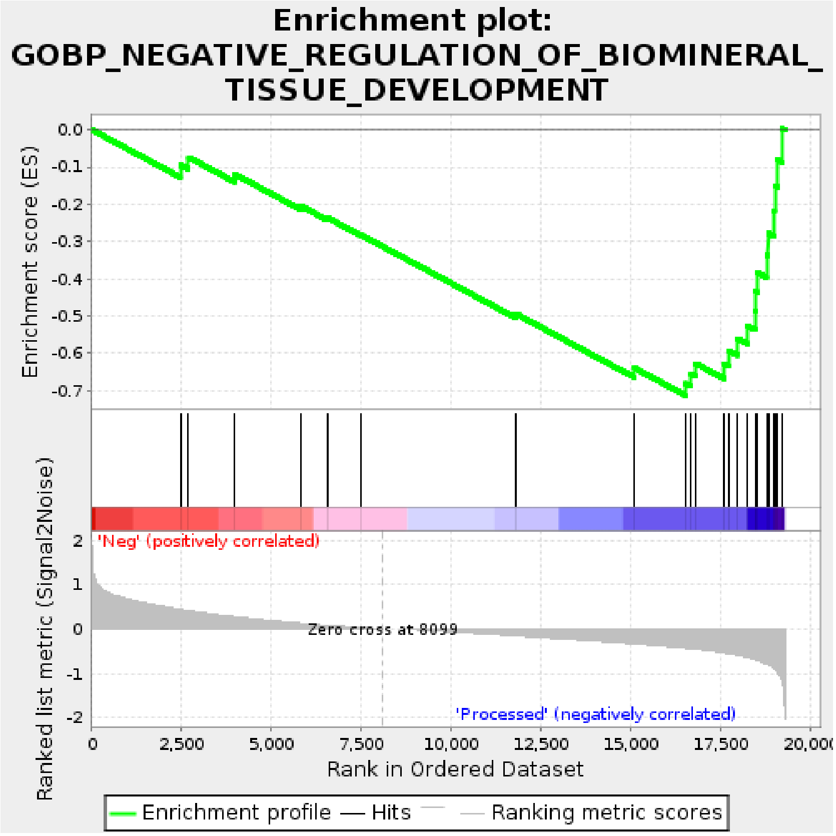
Enrichment plot:
GOBP_NEGATIVE_REGULATION_OF_BIOMINERAL_TISSUE_DEVELOPMENT
Enrichment score (ES)
Ranked list metric (Signal2Noise)
'Neg' (positively correlated)
Zero cross at 8099
'Processed' (negatively correlated)
Rank in Ordered Dataset
Enrichment profile — Hits — Ranking metric scores

(h)

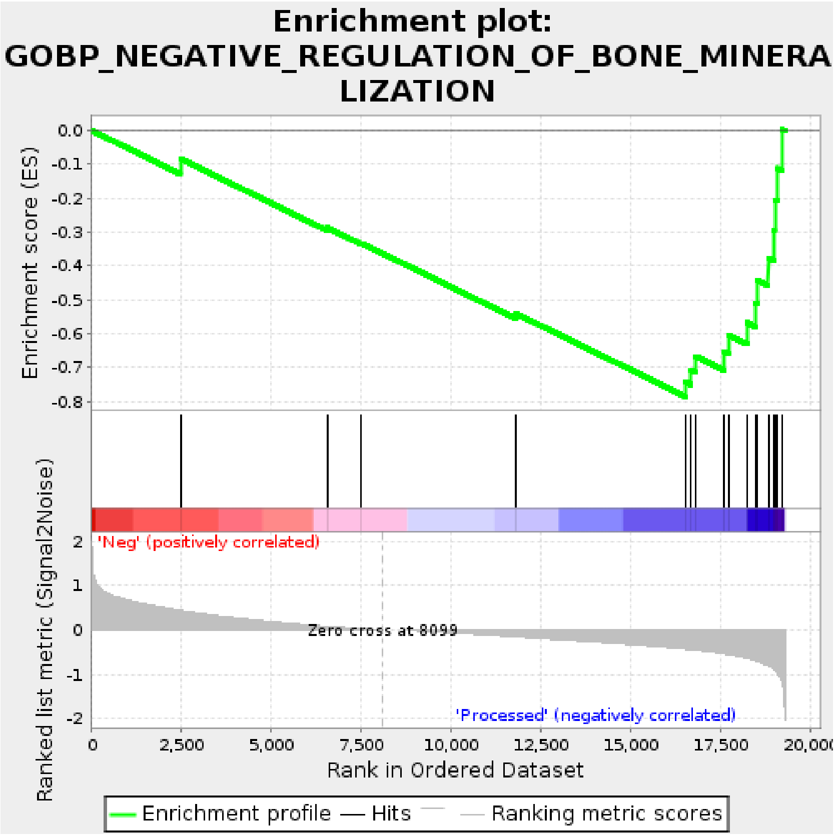

(i)

Supplementary Figure S3. (a) GSEA_Pos_vs_Neg_GO-BP_top1 (|NES|=2.51, FDR q-value<0.05); (b) GSEA_Pos_vs_Neg_GO-BP_top2 (|NES|=2.51, FDR q-value<0.05); (c) GSEA_Pos_vs_Neg_GO-BP_top3 (|NES|=2.50, FDR q-value<0.05); (d) GSEA_Raw_vs_Neg_GO-BP_top1 (|NES|=2.68, FDR q-value<0.05); (e) GSEA_Raw_vs_Neg_GO-BP_top2 (|NES|=2.66, FDR q-value<0.05); (f) GSEA_Raw_vs_Neg_GO-BP_top3 (|NES|=2.63, FDR q-value<0.05); (g) GSEA_Processed_vs_Neg_GO-BP_top1 (|NES|=2.31, FDR q-value<0.05); (h) GSEA_Processed_vs_Neg_GO-BP_top2 (|NES|=2.25, FDR q-value<0.05); (i) GSEA_Processed_vs_Neg_GO-BP_top3 (|NES|=2.25, FDR q-value<0.05).
